# Supplementary material for: Quantification of Gleason Pattern 4 at MRI-Guided Biopsy to Predict Adverse Pathology at Radical Prostatectomy in Intermediate-Risk Prostate Cancer Patients
Source: Cancers (Basel). 2023 Nov 17;15(22):5462. doi: 10.3390/cancers15225462 (PMC10670701; doi:10.3390/cancers15225462)
Supplement: Supplementary file 1 [file cancers-15-05462-s001.zip › cancers-2640329-supplementary.pdf]

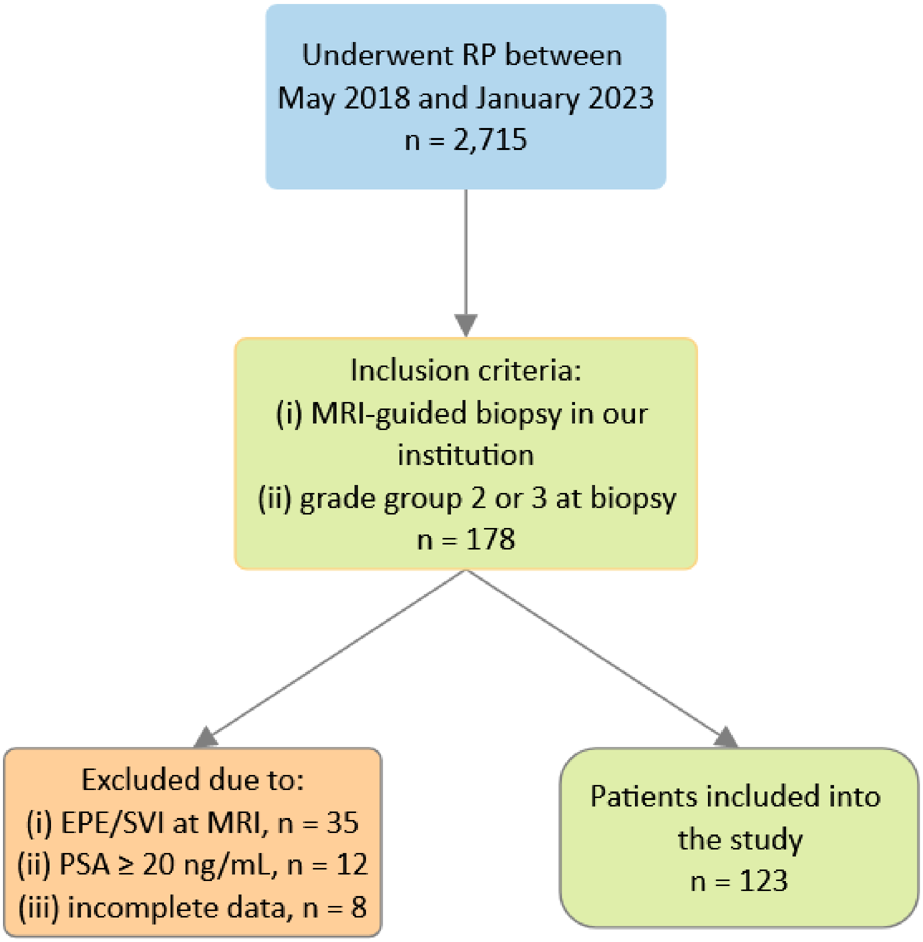

Supplementary Figure S1: Flowchart of patient inclusion and exclusion.

Supplementary Table S1. Associations of patient characteristics and biopsy features with adverse pathology at radical prostatectomy on univariable logistic regression models.

Independent variable			OR, 95% CI	p-value
Age, years			1.04 (0.98-1.10)	0.160
PSA, ng/mL			1.13 (0.99-1.27)	0.060
PIRADS category 5			1.25 (0.55-2.85)	0.592
PV, mL			1.03 (1.00-1.05)	0.059
PSAD, ng/mL^2			1.04 (0.04-24.39)	0.982
Grade group 3 (vs 2)			1.96 (0.80-4.85)	0.143
GP4 amount				
	GP4%cores	OB	1.09 (1.02-1.16)	0.007
		TB	1.02 (0.99-1.04)	0.221
		SB	1.20 (1.03-1.39)	0.021
		WS	1.03 (1.00-1.05)	0.020
	GP4%cancer	OB	1.01 (1.00-1.03)	0.101
		TB	1.02 (1.00-1.03)	0.068
		SB	1.02 (1.00-1.05)	0.101
		WS	1.02 (1.00-1.03)	0.077

GP4mm	OB	1.05 (1.01-1.10)	0.009
	TB	1.06 (1.01-1.10)	0.011
	SB	1.10 (0.91-1.34)	0.312
	WS	1.06 (1.01-1.11)	0.012
PV×GP4		1.27 (1.07-1.51)	0.006
Cribriform pattern present		1.10 (0.51-2.39)	0.807

PSA, prostate-specific antigen; PIRADS, Prostate Imaging Reporting and Data System; PV, prostate volume; PSAD, PSA density; GP4, Gleason pattern 4; OB, overall biopsy; TB, targeted biopsy; SB, systematic biopsy; WS, worst specimen

Supplementary Table S2. Spearman rank correlation analysis for selected patient characteristics and biopsy features.

Variable	GP4%cores, OB	GP4%cores, SB	GP4%cores, WS	GP4mm, OB	GP4mm, TB	GP4mm, WS	PV×GP4
GP4%cores, OB	–	0.25**	0.91***	0.91***	0.89***	0.90***	0.92***
GP4%cores, SB	0.25**	–	0.17[t]	0.34***	0.32***	0.23*	0.25**
GP4%cores, WS	0.91***	0.17[t]	–	0.82***	0.80***	0.83***	0.84***
GP4mm, OB	0.91***	0.34***	0.82***	–	0.98***	0.98***	0.83***
GP4mm, TB	0.89***	0.32***	0.80***	0.98***	–	0.99***	0.80***
GP4mm, WS	0.90***	0.23*	0.83***	0.98***	0.99***	–	0.82***
PV×GP4	0.92***	0.25**	0.84***	0.83***	0.80***	0.82***	–

PV, prostate volume; GP4, Gleason pattern 4; OB, overall biopsy; TB, targeted biopsy; SB, systematic biopsy; WS, worst specimen

[t] $0.05 \leq$ p-value < 0.10

* $0.01 \leq$ p-value < 0.05

** $0.001 \leq$ p-value < 0.01

*** p-value < 0.001
